# Aminic Organoselenium Compounds: Promising Antioxidant Agents

**DOI:** 10.1021/acsomega.5c04072

**Published:** 2025-09-15

**Authors:** Babli Chhillar, João M. Brito, Nikhil Sodhi, Climei R. Cabreira, Sumbal Saba, Jamal Rafique, Vijay P. Singh

**Affiliations:** † Department of Chemistry & Centre of Advanced Studies in Chemistry, 29750 Panjab University, Sector-14, Chandigarh 160 014, India; ‡ LabSO, Instituto de Química−IQ, Universidade Federal de Goiás−UFG, Goiânia 74690-900, GO, Brazil; § Instituto de Química−INQUI, Universidade Federal do Mato Grosso do Sul−UFMS, Campo Grande 79074-460, MS, Brazil

## Abstract

Significant research interest has been devoted to organoselenium compounds due to their diverse biological activities, particularly their antioxidant properties, which mimic those of glutathione peroxidase (GPx) enzymes. While previous reviews have explored the broad spectrum of organoselenium chemistry and their applications, this review focuses specifically on aminic organoselenium compounds as next-generation GPx mimics and radical-trapping antioxidants. This work presents a comprehensive analysis of their catalytic mechanisms and structure–activity relationships (SAR) and the methodologies employed to evaluate their antioxidant efficacy. Unlike earlier reviews, this work emphasizes the critical role of aminic moieties in enhancing GPx-like activity and dual functionalities (peroxide decomposition and radical quenching). Key topics include the design principles of aminic organoselenium compounds, their performance relative to classical antioxidants like ebselen and α-tocopherol, and their promising use in developing treatments for conditions linked to oxidative stress. By consolidating advancements from the past two decades, this review connects existing research areas and suggests valuable pathways for future studies in developing multifunctional organoselenium antioxidants.

## Graphical abstract

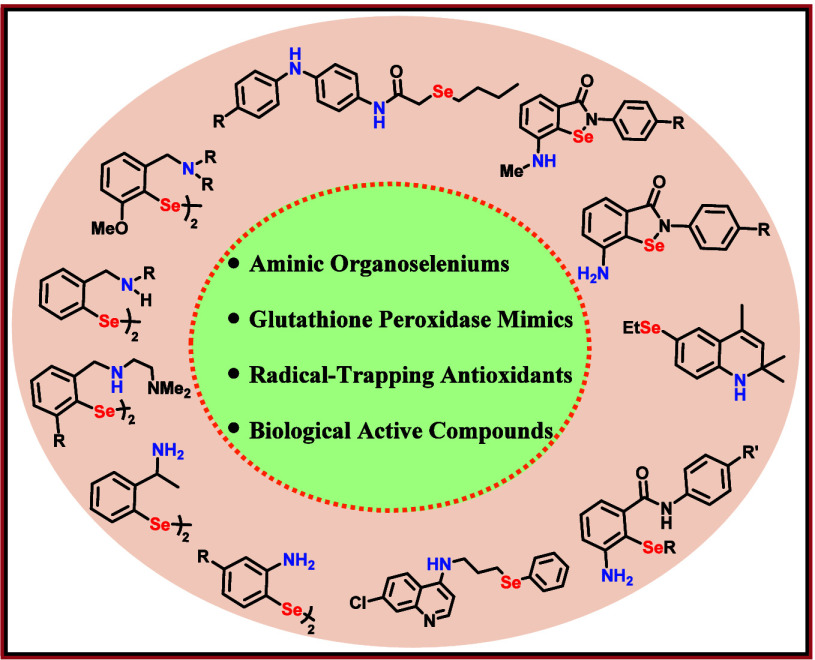

## 1 Introduction

In 1817, Jons Jacob Berzelius, a Swedish chemist, successfully extracted selenium (Se) from the lead chambers of a sulfuric acid factory.^1^ Noting its similarity to tellurium, which was named for the Roman earth goddess Tellus, Berzelius chose to name the new element after Selene, the Greek goddess of the moon.^1c^ The first organoselenium compound was diethyl selenide, synthesized by Lowig in 1836.^2^ The initial phase of organoselenium chemistry focused on the synthesis of basic structures, including selenols (RSeH), selenides (RSeR), and diselenides (RSeSeR); however, these early compounds proved to be unstable and difficult to isolate in their pure form. However, organoselenium chemistry became more famous in the 1970s after the reorganization of several useful reactions and a variety of novel structures, and since then has been extensively used in the material science and catalyst development.^3,4^ These compounds display a spectrum of fascinating biological properties, which are largely attributed to their activity against oxidative stress, inflammation, Alzheimer’s disease, and cancer.^2,5,6^ Se is a vital element found in key biological macromolecules, specifically, within the proteins of diverse organisms. In these selenoproteins, the Se atom forms a covalent bond with carbon, leading to the formation of an organoselenium molecule. Numerous specific selenoproteins, each containing at least one selenocysteine residue are prevalent in animals, with a particular abundance observed in vertebrates.^6e^

Research into Se is driven by its essential role in selenoproteins, which are vital for mitigating oxidative damage from excessive reactive oxygen species (ROS). The identification of selenocysteine (SeCys), the redox-active, Se-containing 21st amino acid encoded genetically within selenoproteins, represents a critical finding, as it plays an essential antioxidant role in human pathological processes.^7,8^ The amino acid selenocysteine (SeCys) is encoded by the UGA codon and inserted cotranslationally during the protein synthesis.^9,10^ SeCys has a molecular structure resemblance to cysteine, where the sulfur atom is replaced by selenium. The selenolate anion, as the conjugate base of SeCys, exhibits greater stability compared to cysteine thiolate.^11^ Moreover, being more acidic (p*K*_a_ 5.2) than thiols (RSH; p*K*_a_ 8.5), selenol (RSeH) readily dissociates at physiological pH, potentially explaining its significant biological reactivity.^12,13^ Among the 25 selenoproteins, glutathione peroxidase (GPx) enzymes are important selenoenzymes having SeCys at their active site.^14^ The active site of GPx enzymes features a catalytic triad where a SeCys residue is stabilized by interactions with tryptophan (Trp) and glutamine (Gln).^15^ The selenol moiety of the SeCys residue is stabilized by H-bonding interactions between the amido group of a Gln residue and the imino group of a Trp residue. These interactions are responsible for the enzymatic activity of the GPx enzymes. These scavenge peroxides by catalyzing the reduction of hydrogen peroxide (H_2_O_2_) to water (H_2_O) and organic peroxides (ROOH) to alcohols (ROH), using the abundant reducing agent glutathione (GSH).^14^

The GPx-family comprises four selenium-dependent enzymes (GPx1–4), each characterized by a catalytically essential selenocysteine (SeCys) residue. Among these, GPx1 is the most ubiquitous isoform. GPx2 is predominantly expressed in the gastrointestinal tract, GPx3 is a secreted extracellular enzyme, and GPx4 is unique for its ability to reduce phospholipid hydroperoxides.^16^ GPx4 has a unique ability to protect cells from lipid peroxidation by reducing lipid hydroperoxides into lipid alcohols using a stoichiometric amount of GSH as a coantioxidant.^17^ GPx enzymes operate via a catalytic cycle centered on a selenocysteine residue. The Se atom in this residue undergoes redox changes, with the catalytically active species being selenol (GPxSeH) (Scheme 1).

**Scheme 1:**
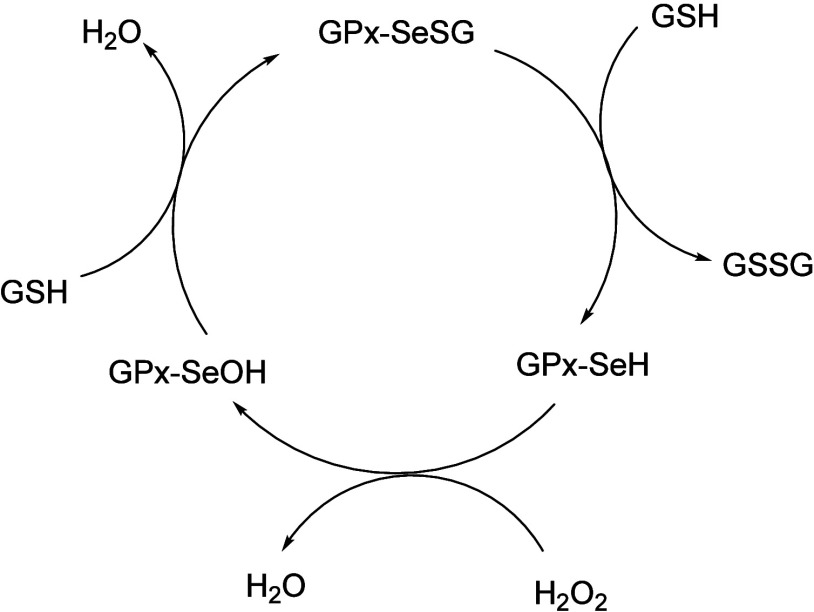
Illustration of the Catalytic Cycle Employed by GPx Enzymes

GPx-SeH reduces the peroxides into selenenic acid (GPx-SeOH). This acid then reacts with GSH to produce a selenyl sulfide (GPx-SeSG), which is subsequently reduced back to the active selenol by a second GSH molecule. Glutathione reductase (GR) serves to reduce the oxidized GSH, i.e., GSSG using nicotinamide adenine dinucleotide phosphate (NADPH) as a cofactor.^6,18–20^

Peroxides and other ROS serve as essential components that, upon release from neutrophils, are involved in redox signaling processes and provide a defense mechanism against pathogenic agents.^21^ The negative aspect of ROS is their involvement in promoting oxidative stress, a phenomenon intricately associated with the pathogenesis of many degenerative disorders that encompass various conditions, including neurological ailments (e.g., Alzheimer’s and Parkinson’s), metabolic diseases like diabetes, and life-threatening illnesses such as cancer, cardiovascular disease, and stroke.^22–24^ In a normal physiological state, the stability of the cellular redox environment is dependent on the coordinated function of multiple antioxidant mechanisms. In humans, there are several antioxidants available that can stabilize the free radicals and prevent chain reactions.^25^ One significant lipophilic antioxidant in the human body is vitamin E, comprising several compounds, among which α-tocopherol (α-TOH) is the most prominent, known as nature’s prime ROS scavenger.^26^ It can quench two peroxyl radicals before converting to the nonradical product form in the presence of ascorbic acid (vitamin C), a water-soluble antioxidant that lacks a phenolic moiety. Vitamin C exists in ascorbate at physiological pH, which is responsible for the regeneration of α-TOH at the aqueous-lipid interface in the cell membrane. Apart from phenolic radical-trapping antioxidants (RTAs), aromatic amine-based compounds, such as diphenylamines (Ar_2_NH), are also commonly used RTAs in the petroleum-derived products.^27^ Ar_2_NH can inhibit the hydrocarbon autoxidation via hydrogen atom transfer (HAT) from the secondary amino group to peroxyl radicals (ROO•).^28^ In amines, 4,4′-dioctyldiphenylamine is an industrial standard having *k*_inh_ = 1.8 × 10^5^ in PhCl at 37 °C.^29^ At elevated temperatures, a much greater stoichiometric factor has been reported. Diarylamines react with ROO• by formal HAT from the N–H group, which has a bond dissociation energy (BDE) of the N–H bond, i.e., BDE_N–H_ ∼ 82 kcal/mol;^30^ which is comparatively lower than BDE_O–H_ of phenol ∼87.2 kcal/mol,^31^ which is driven by their slightly enhanced activity. Substitution at 4 and 4′-positions by electron-donating groups in Ar_2_NH also weakened the BDE_N–H_ and improved the antioxidant activities (e.g., alkoxy and dialkylamino groups demonstrated stabilization energies of 4.0 and 6.3 kcal/mol, respectively).^30^

Since the finding of SeCys at the active site of the GPx, researchers are increasingly developing diverse organoselenium compounds exhibiting biological activities, including antioxidant properties. Among the reported organochalcogens, the first antioxidant was a heterocyclic aromatic ring containing Se–N, ebselen (2-phenyl-1,2-benzoisoselenazol-3-(2*H*)-one, **1**), discovered by Sies and Wendel, that could mimic the GPx enzyme.^32,33^ The initially proposed catalytic cycle for the GPx-like activity of **1** involves the attack of thiols to cleave the Se–N bond to produce selenyl sulfide intermediate **2**. Additional reduction of the Se–S bond through excess thiol compounds produces the catalytically active selenol intermediate, which undergoes rapid oxidation by hydroperoxide substrates to form selenenic acid **4**. The selenenic acid **4** underwent reaction with thiol to regenerate selenyl sulfide **2**. However, this mechanism is expected when GSH is used as a thiol. In the case of aryl thiols and benzyl thiols, **1** showed significantly reduced activity for H_2_O_2_ reduction.^34–37^ A revised mechanism has been proposed by Mugesh and co-workers, as shown in Scheme 2.^38,39^

**Scheme 2:**
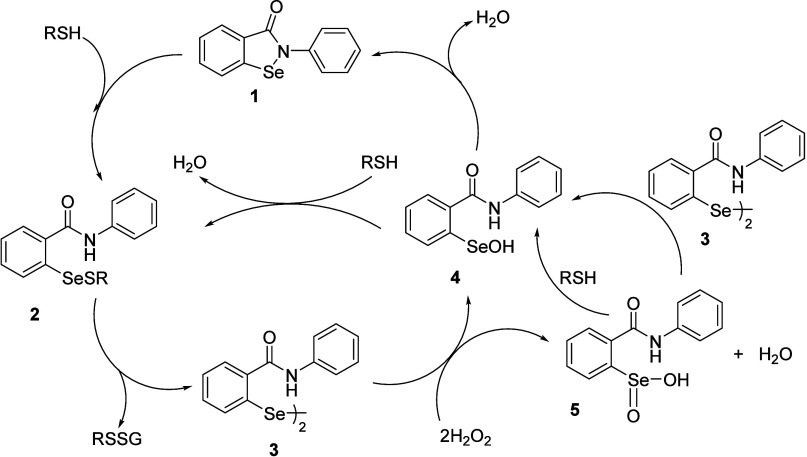
Catalytic Cycle Demonstrating the GPx-like Activity of **1**

In the absence of thiol, **1** underwent overoxidation to produce seleninic acid intermediate **5**.^38^ The reaction of **5** with an excess amount of thiol produced selenyl sulfide **2**. The major pathway for the GPx-like activity of **1** involved the disproportionation of intermediate **2** to form **3**. This diselenide subsequently reacted with H_2_O_2_, yielding a mixture of selenenic acid **4** and seleninic acid **5**. Selenenic acid **4** then reacted with PhSH to form selenenyl sulfide **2** or underwent cyclization to regenerate **1**. Meanwhile, seleninic acid **5** was converted to **4** by reacting with either PhSH or diselenides **3**. The formation of diselenide **3** was identified as the rate-determining step of this catalytic cycle. After the discovery of ebselen, many more organoselenium compounds, such as Se–N^40,41^ heterocycles and Se–O^42^ heterocycles, selenides,^43^ diselenides,^44^ spirocyclic,^45,46^ and other compounds, have been found to exhibit GPx-like enzymatic behavior.^47^ In the past few years, aminic organoselenium compounds have been investigated as potential next-generation GPx-mimics and RTAs. Herein, we comprehensively cover the above aspects of organoselenium aminic compounds obtained in the last 18 years. This review will serve as a valuable guide for future research on pharmacological applications of organoselenium aminic compounds.

2

### 2.1 Methods for the Evaluation of GPx-like Activity

#### 2.1.1 Enzymatic Method/Coupled-Reductase Assay^48^

Initially, Wilson et al. pioneered the application of this technique in the analysis of GPx-like activity of organoselenium compounds. The enzymatic activity of compounds was assessed by employing hydrogen peroxide (H_2_O_2_) as the substrate in conjunction with GSH. GR, facilitated by NADPH as a cofactor, was employed to catalyze the reduction of oxidized GSH (eqs 1-3).

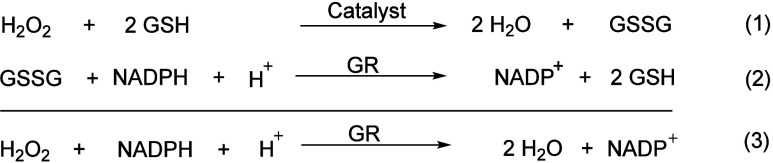

1



The initial reaction rates (*v*_0_), determined by monitoring the NADPH consumption at 340 nm, quantified the GPx-like activity. The synthesized compounds’ relative activities were benchmarked against natural GPx enzymes. An assay solution containing potassium phosphate buffer, EDTA, GSH and GR was prepared with the test compound. The reaction was then initiated by adding H_2_O_2_.

#### 2.1.2 Thiophenol Assay as Alternative of GSH^49^

Iwaoka and Tomoda utilized this approach using benzenethiol (PhSH) as an alternative to GSH. In this assay, the GPx-mimetic catalytic reaction was started by introducing an excess of H_2_O_2_ into a methanolic solution of PhSH (*C*_0_) and a selenium-based catalyst. Progress was tracked via UV–vis spectroscopy by observing the absorbance at 305 nm, which increases due to the production of diphenyl disulfide (PhSSPh). The initial hydrogen peroxide reduction rates (ν_0_) were calculated from the absorbance change. The large molar extinction coefficient of PhSSPh at this wavelength (ε = 1.24 × 10^3^ M^–1^ cm^–1^), compared to that of PhSH (ε = 9 M^–1^ cm^–1^), allowed the concentration of remaining PhSH (C) to be calculated with the equation *C* = (ε_1_ *C*_0_–2*a*)/(ε_1_ – 2ε_2_) ≈ *C*_0_–2*a*/ε_1_, where ε_1_ and ε_2_ represent the molar extinction coefficients of PhSSPh and PhSH, respectively. The corresponding reaction equation is shown below (eq 4):

#### 2.1.3 HPLC Method^49,50^

In this assay, a reversed-phase HPLC method was employed to track the production of PhSSPh. Conversion rates were assessed by measuring the half-life (*t*_1/2_) for PhSH consumption, derived from analysis of the chromatographic peak areas over time.

$$2\mathrm{PhSH}+H_{2}O_{2}\overset{\mathrm{Se}\mathrm{catalyst}}{\to }2H_{2}O+\mathrm{PhSSPh}$$ 4

#### 2.1.4 DTT^red^/DTT^ox^ NMR Assay

The GPx-mimetic activity of organoselenium compounds can be assessed via a reported NMR-based assay .^51,52^ This method monitors the catalytic reduction of H_2_O_2_, with dithiothreitol (DTT^red^) serving as the sacrificial thiol. Regeneration of the catalyst is tracked by measuring the formation of the disulfide (DTT^red^) by ^1^H NMR in CD_3_OD or D_2_O over time. The reaction is described by the following eq (eq 5):



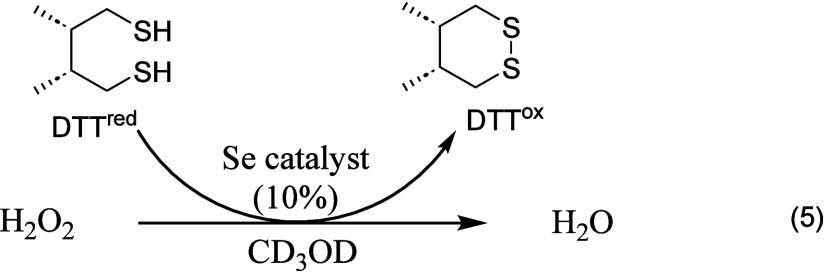

5


### 2.2 Methods for the Evaluation of RTA Activity

#### 2.2.1 HPLC Lipid Peroxidation Assay^53^

Engman et al. have often used azo-initiated peroxidation of linoleic acid or derivatives for analyzing the antioxidative properties of organochalcogen compounds. A recently published account details the experimental methodology used to determine inhibition times (*T*_in*h*_) and inhibited peroxidation rates (*R*_in*h*_) in the azo-initiated peroxidation of linoleic acid within a two-phase system. In the modified system, a suitable water-soluble coantioxidant was present in the water phase, which in the lipid phase was able to regenerate the active antioxidant. In a typical procedure, the antioxidant was assessed by vigorously stirring a mixture of linoleic acid and the test compound in chlorobenzene at 42 °C with an aqueous *N*-acetylcysteine (NAC) solution. The peroxidation of linoleic acid was initiated at 42 °C with 2,2′-azobis­(2,4-dimethylvaleronitrile) (AMVN), equipped with an autoinjector in the organic phase. The peroxidation progress was tracked via HPLC by measuring UV absorption at 234 nm, characteristic of conjugated dienes. Catalyst efficiency was assessed by calculating the inhibited peroxidation rate (*R*_in*h*_) from absorbance-versus-time data using a least-squares analysis. The duration of the inhibited phase (*T*_in*h*_), measured over 300 min, was determined graphically from the intersection point of the lines representing the inhibited and uninhibited reaction rates.

#### 2.2.2 2,2-Diphenyl-1-picrylhydrazyl (DPPH) Assay

Shaaban et al. delineated an alternative approach for evaluating organoselenium compounds’ radical scavenging activity via the DPPH method.^54^ This approach measures antioxidant potential by the reduction of the purple DPPH• radical to a colorless product, monitored by a decline in absorbance at 517 nm.

#### 2.2.3 2,2′-Azino-bis­(3- ethylbenzothiazoline-6-sulfonic acid) (ABTS) Assay

Shaaban et al. introduced another approach for evaluating radical scavenging activities of organoselenium compounds.^55^ The radical-scavenging activity of the organoselenium compounds was determined by measuring their ability to decolorize the ABTS**•** (2,2′-azino-bis­(3-ethylbenzothiazoline-6-sulfonic acid) radicals, monitored by a decrease in absorbance at 734 nm.

## 3 Aminic Organoselenium Antioxidants

### 3.1 Aminic Organoselenium Antioxidants Reported by Mugesh et al.

Mugesh and coauthors showed the effect of various amino groups (R_2_N–, RR̀N–, RNH–, H_2_N−) on the GPx-like activity of a variety of diaryl diselenides. Initially, they reported the synthesis and GPx-like activity of *tert-*amino-substituted diselenides **6a**–**c** and **7a**–**c** (Figure 1).^50b^

**Figure 1:**
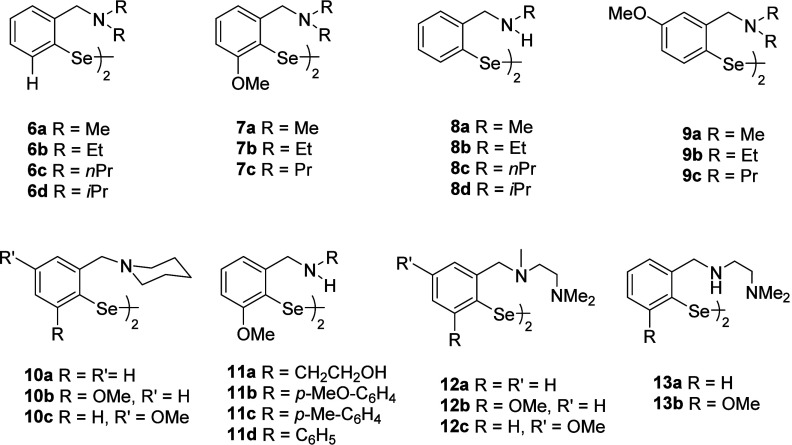
Benzylamine-based organoselenium compounds.

The GPx-mimetic activity was evaluated using HPLC.^50^ The assays employed H_2_O_2_, cumene hydroperoxide (Cum-OOH), or *tert-*butylhydroperoxide (*t-*BuOOH) as the substrates and PhSH as the cosubstrate. The reaction progress was tracked by quantifying PhSSPh formation, and the half-life (*t*_1/2_) for 50% PhSH conversion was derived from the peak areas measured over time. (Table 1).

**Table 1:** Peroxide Reduction Half-Lives (*t*_1/2_) with PhSH Catalyzed by **6a**–**c** and **7a**–**c** at 22 °C

	*t*_1/2_ values (min)^a^		
catalyst	H_2_O_2_	Cum-OOH	^*t*^Bu-OOH
**6a**	19.2	16.5	24.4
**6b**	19.9	17.6	35.2
**6c**	22.7	24.3	49.7
**7a** ^b^	3.8	2.6	6.3
**7b**	12.5	9.1	29.0
**7c**	13.2	12.1	35.2

a Conditions for assay: The reactions were carried out at 22 °C in MeOH. [Catalyst (10.0 mm, except **7a**), thiophenol: (1.0 mm), R_2_O_2_ (2.0 mm)].

b 5 mm concentration of the catalyst was used, when the conversion was too fast to be measured at 10 mm concentration.

Diselenides **6a**–**c** presented higher GPx-like activities compared to compound **1** in all three peroxide systems used. The higher GPx-like activity of diselenides **6a**–**c** is probably due to the presence of the dialkylamino functionality that could generate a more reactive selenolate by deprotonating the selenol. Interestingly, a significant enhancement in the activity was observed when the proton at the 6-position in compounds **7a**–**c** was replaced by methoxy substitutents.^50b^ The *tert*-amino group in diselenides **6a**–**c** and **7a**–**c** served as a strong base to deprotonate the thiol, generating a more nucleophilic thiolate anion for attack on the Se center.

Subsequently, the GPx-like activity was also documented for *sec-*amino-substituted diselenides **8a**–**d**.^56^ The GPx-like activity of these diselenides was studied by using PhSH (thiol cofactors) and H_2_O_2_, Cum-OOH, and *t*BuOOH as substrates, measured by HPLC,^50^ and benchmarked against *N,N*-dialkyl derivatives **6a**–**d**. For each reaction, the quantity of PhSSPh generated was measured, and the half-life (*t*_1/2_ values) corresponding to 50% conversion of PhSH was calculated (Table 2).

**Table 2:** PhSH-Mediated Peroxide Reduction Catalyzed by **6a**–**d** and **8a**–**d**

	*t*_1/2_ values (min)^a^		
catalyst	H_2_O_2_	Cum-OOH	^*t*^Bu-OOH
control	2905	1130	798
**6a**	67	72	49
**6b**	91	100	63
**6c**	292	206	116
**6d**	272	197	81
**8a**	37	36	17
**8b**	66	58	24
**8c**	74	80	27
**8d**	118	100	40

a Conditions for assay: The reactions were carried out at 22 °C in MeOH. Catalyst: 5.0 mm; thiophenol: 1.0 mm; R_2_O_2_: 2.0 mm. The control reactions were carried out under similar conditions in the absence of Se catalyst.

It has been found from the data that the GPx-like activities of diselenides **8a**–**d** (*sec-*amine based) were significantly greater than those of the corresponding *tert-*amine derivatives **6a**–**c** in all three peroxides. Furthermore, the investigation of the reduction of peroxides catalyzed by amino-substituted diselenides **6a**–**d** and **8a**–**d** was also conducted by utilizing GSH (cosubstrate). The initial rate of the reaction (*v*_0_) was determined using a coupled-reductase assay in the presence and absence of Se compounds.^48^ Interestingly, the *sec-*amine-based diselenides **8a**–**d** presented higher activities compared to their *tert-*amine-based conterparts **6a**–**d**. Notably, the *n-*Pr and *i-*Pr derivatives **8c** and **8d** exhibited approximately 8–18 times greater activity as compared to their respective *n*-Pr_2_N-derivative **6c** and *i*-Pr_2_N-derivative **6d**. This disparity is likely attributed to the *sec*-amino group in **8c** and **8d** being more polar, thereby increasing the compounds’ solubility in the assay buffer.

Thereafter, the synthesis of a series of 4-methoxy group containing *tert-*amine-based diselenides **9a**–**c** and other related diselenides **10a**–**c** has been reported, and GPx-like activity was compared with compounds **6a**–**c** and the corresponding 6-methoxy-substituted compounds **7a**–**c**.^57^ The GPx-like activity of these diselenides was investigated using PhSH (to mimic GSH), alongside the peroxide substrates H_2_O_2_, Cum-OOH, and ^*t*^BuOOH, using the HPLC method.^50^ The time needed for 50% consumption of PhSH (to form PhSSPh), defined as *t*_1/2_, was measured (Table 3).

**Table 3:** PhSH-Mediated Peroxide Reduction Catalyzed by **6a**–**c**, **7a**–**c**, **9a**–**c,** and **10a**–**c**

	*t*_1/2_ values (min)^a^		
catalyst	H_2_O_2_	Cum-OOH	^*t*^Bu-OOH
control	512.0 ± 20.2	781.0 ± 41.0	471.0 ± 33.7
**6a**	18.3 ± 0.1	23.9 ± 3.4	22.4 ± 1.5
**6b**	19.8 ± 0.7	35.5 ± 4.6	24.3 ± 0.8
**6c**	27.1 ± 0.5	47.3 ± 11.2	23.8 ± 2.2
**7a** ^b^	5.8 ± 0.1	12.5 ± 1.2	4.9 ± 0.2
**7b**	13.2 ± 0.3	37.8 ± 5.1	11.9 ± 1.1
**7c**	15.7 ± 0.6	40.3 ± 2.9	14.1 ± 0.9
**9a**	14.5 ± 1.2	12.5 ± 0.8	11.6 ± 0.1
**9b**	16.8 ± 1.7	16.9 ± 0.6	12.9 ± 0.6
**9c**	23.9 ± 2.1	17.3 ± 0.9	15.0 ± 1.3
**10a**	53.7 ± 3.8	60.4 ± 5.1	46.5 ± 2.1
**10b**	39.1 ± 4.3	45.0 ± 1.5	13.0 ± 1.4
**10c**	24.6 ± 3.1	28.1 ± 3.7	16.9 ± 3.1

a Conditions for assay: The reactions were carried out at 22 °C in MeOH. Catalyst: 10.0 mm (except **7a**); thiophenol: 1.0 mm; R_2_O_2_: 2.0 mm. The control reactions were carried out under similar conditions in the absence of Se compounds.

b Catalyst: 5.0 mM.

Diselenides **7a**–**c** having 6-methoxy substituents were found with improved GPx-like activity compared to diselenide **6a**–**c**. The introduction of a methoxy substituent improves catalytic activity by preventing thiol exchange reactions at the Se atoms of the selenyl sulfide intermediate.^50b^ Also, it was observed that the GPx-like activity of diselenides **9a**–**c** has been found to be higher than that of diselenides **6a**–**c** due to the electronic effect of the methoxy substituent. A substantial improvement in the GPx-like activity of diselenide **10c** was also noted when a methoxy substituent was introduced. The reduced activity of **10b**, relative to **10c**, is likely attributable to its *N*-cyclohexane and methoxy substituents around the Se atom that created steric hindrance. Diselenides **9a**–**c** were also evaluated for their ability to catalyze peroxide reduction with GSH.^57^ It was observed that 4-methoxy-substituted diselenide **9a**–**c** exhibited higher GPx-like activity than 6-methoxy-substituted diselenides **7a**–**c,** as the methoxy substituents in diselenides provided steric hindrance around the Se atom when nucleophile GSH attacked at the Se center.

Subsequently, *sec-*amine-based diselenides with 6-methoxy substituent **11a**–**d** have also been reported, and their GPx-like activity was evaluated using a coupled-reductase assay.^48,58^ It was found that all of these diselenides exhibited significantly higher GPx-like activity compared to compound **1**. Another series of diselenides, **12a**–**c** and **13a**–**b,** containing an additional amino group on the benzylic nitrogen atom, has been synthesized.^59^ The GPx-like activity of compounds **12a**–**c** and **13a**–**b** was studied using PhSH as the thiol cofactor and three different peroxides H_2_O_2_, Cum-OOH, or *t-*BuOOH as substrates using the reverse-phase HPLC method,^50^ and their activities were compared to that of the corresponding diselenides **6a**, **7a**, **8a,** and **9a**. A comparison of the *t*_1/2_ values using PhSH as the cofactor clearly indicates that the activity is enhanced significantly in the presence of an additional amino group (Table 4).

**Table 4:** PhSH-Mediated Peroxide Reduction Catalyzed by **6a**, **7a**, **8a**, **9a, 12a-c,** and **13a**–**b**

	*t*_1/2_ values (min)^a^		
compound	H_2_O_2_	^*t*^Bu-OOH	Cum-OOH
**6a**	18.1 ± 2.2	17.3 ± 3.5	23.9 ± 3.5
**7a** ^b^	4.4 ± 1.0	5.1 ± 1.2	11.0 ± 2.1
**8a** ^b^	7.8 ± 0.5	8.9 ± 1.2	12.9 ± 2.1
**9a**	14.5 ± 2.3	12.2 ± 2.0	23.7 ± 3.5
**12a**	10.6 ± 1.5	13.1 ± 2.1	22.9 ± 4.2
**12b** ^b^	5.9 ± 0.5	7.3 ± 1.3	14.3 ± 1.5
**12c**	12.2 ± 1.2	11.1 ± 1.5	17.2 ± 2.5
**13a** ^b^	5.4 ± 1.3	7.4 ± 1.6	21.6 ± 3.8
**13b** ^b^	2.8 ± 0.2	4.6 ± 1.5	8.6 ± 2.3

a Conditions for assay: The reactions were carried out at 22 °C in MeOH. Catalyst: 10.0 mm (except **7a**, **8a**, **12b**, **13a**–**b**); thiophenol: 1.0 mm; R_2_O_2_: 2.0 mm. The control reactions were carried out under similar conditions in the absence of Se compounds.

b Catalyst: 5.0 mM.

Introduction of an additional amino group significantly enhanced the GPx-like activity of diselenides **12a**–**b** and **13a**–**b**. Intriguingly, the GPx-like activity of diselenide **13b** was found to be 1.5 times higher than that of diselenide **7a**.

### 3.2 Aminic Organoselenium Antioxidants by Braga and Rocha et al.

Over recent years, Braga and Rocha research groups were involved in the synthesis and screening of novel organoselenium compounds designed to be highly effective GPx enzyme mimics. Earlier, they reported the chiral ephedrine derivatives, diselenide **14** and selenides **15a**–**b,** and their GPx-like activity using the thiophenol assay­(Figure 2).^49,60^

**Figure 2:**
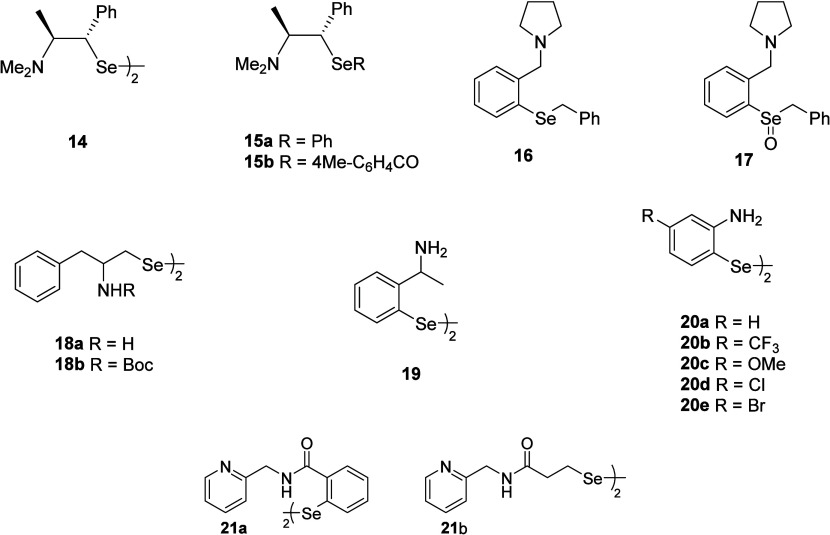
Aminic organoselenium compounds.

Time required by catalysts **14** and **15** to decrease the concentration of the PhSH to half (*T*_50_) is presented in Table 5. This study used diphenyl diselenide (Ph_2_Se_2_), a well-known GPx mimic, as a reference compound for comparison.

**Table 5:** GPx-like Activity of Catalysts **14**–**15** and Ph_2_Se_2_

catalyst^a^ ^,^ ^b^	*T*_50_ values (min)^c^
Ph_2_Se_2_	187.28 ± 7.53
**14**	16.33 ± 1.30
**15a**	244.27 ± 25.05
**15b**	48.75 ± 5.47

a Values of *T*_50_ were corrected for the uncatalyzed background reaction.

b MeOH (1 mL); catalyst: **14–15** and Ph_2_Se_2_ (0.05 mM); thiophenol (5 mM); H_2_O_2_ (10 mM).

c *T*_50_ (minutes) is the time for thiol concentration to drop by half after H_2_O_2_ is added.

Diselenide **14** promoted a 50% reduction in the PhSH concentration that occurred in only 16.3 min. Selenide **15a** showed poorer catalytic activity in this set of experiments. The activity of ephedrine derivative **15b** is substantially impacted by the in situ formation of a selenolate from its labile selenoester functionality. Furthermore, catalyst **15b** demonstraed catalytic performance approximately four times higher than standard Ph_2_Se_2_.

The initial rates for the formation of PhSSPh were observed for the H_2_O_2_ oxidation of PhSH catalyzed by **1** and **16–17** (Table 6).^61^

**Table 6:** GPx-like Activities of Catalysts **1** and **16**–**17** ^a^

catalyst	GPx-like activity *v*_0_ (μM·min^–1^)^b^
**1**	17.18 ± 0.17
**16**	19.29 ± 0.19
**17**	51.77 ± 0.18

a Conditions for assay: H_2_O_2_ (final concentration = 10.4 mM in MeOH), thiophenol: (final concentration = 10.0 mM in MeOH), and Se catalyst (final concentration = 10.0 μM in MeOH).

b Values of *v*_0_ were adjusted for the background reaction and are presented as mean ± SD (*n* = 3).

The GPx-like activity of organoselenium compounds **18a**–**b** having nonbonding nitrogen interaction was evaluated using the thiolphenol assay^49^ and compared with Ph_2_Se_2_.^62^ The proximity of an amino group residue to the Se atom may facilitate a weak interaction, stabilizing the active selenolate. Compound **18a** showed higher GPx-like activities compared to Ph_2_Se_2_ (where nonbonding interactions are not present) and compound **18b** (where nonbonding interactions are blocked with bulky groups).

In the next, the GPx-like activity of diselenide **19** was evaluated using ^*t*^BuOOH and H_2_O_2_ as peroxides in the thiophenol assay^49^.^63^ It was observed that diselenide **19** exhibited GPx-like activity 2 times higher than that of Ph_2_Se_2_.

Aniline-derived diselenides **20a**–**e** have been reported with significant GPx-like antioxidant activities.^64^ The catalytic parameters Michaelis–Menten constant (*K*_m_), catalytic constant (*k*_cat_), and the catalytic efficiency (η) were determined for all catalysts as summarized in Table 7.

**Table 7:** GPx-like Activity of Catalysts **20a**–**e**

compounds	*K*_m_ (mol L^*–*1^)	*k*_cat_ (min^*–*1^)	η (L mol^*–*1^ min^*–*1^)
**1**	0.00170	0.422	248.65
Ph_2_Se_2_	0.00114	0.601	527.78
**20a**	0.00134	0.446	333.40
**20b**	0.00105	1.185	1128.57
**20c**	0.00187	0.470	251.51
**20d**	0.00088	0.918	1044.19
**20e**	0.00081	0.405	500.11

With a *para-*CF_3_ group, diselenides **20b** showed 5 times higher activity than compound **1** and was twice as active as Ph_2_Se_2_. These results suggest that stronger electron-withdrawing groups in the *para* position enhance the catalytic efficiency of aniline-derived diselenides. This effect is attributed to the amino group, which hydrogen-bonds to Se to stabilize the selenolate intermediate as a zwitterion.

Diselenide **21a**, which exhibits nonbonding interactions, has demonstrated significant GPx-like activity (4.66-fold higher than Ph_2_Se_2_) and potent lipid peroxidation inhibition.^65^ However, replacement of the aromatic system with an aliphatic structure of equivalent carbon length (diselenide **21b**) resulted in reduced GPx-mimetic activity,^65,66^ underscoring the critical role of aryl-diselenide moieties in catalytic efficiency.

### 3.3 Aminic Organoselenium Antioxidants by Shaaban et al. and Alves et al.

Shaaban et al. described the synthesis of compounds **22–23** (Figure 3).^67^ The radical scavenging antioxidant activity was found to be comparable to vitamin C in the DPPH and ABTS assays.^54,55^

**Figure 3:**
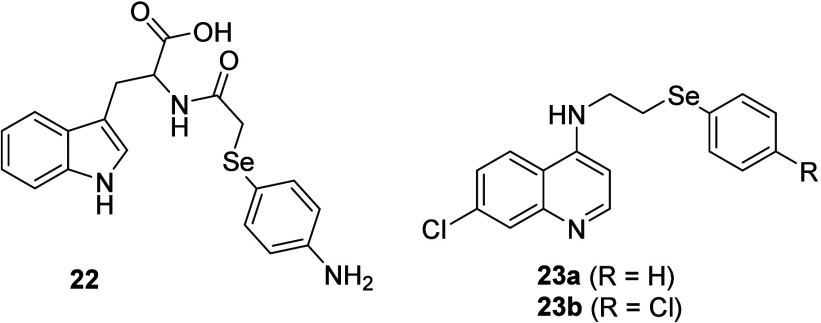
Tryptophan and quinoline organoselenium derivatives **22** and **23**.

Alves et al. reported Se-containing tryptophan and quinoline derivatives **22** and **23** with their excellent radical scavenging antioxidant activity in the DPPH and ABTS assays, respectively.^68^

### 3.4 Aminic Organoselenium Antioxidants by Iwaoka et al.

A series of water-soluble selenides **24–27** containing amine groups have been developed (Figure 4).^69^

**Figure 4:**
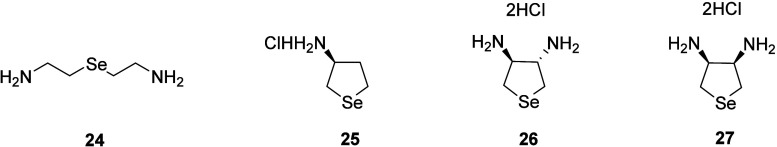
Selenide **24** with cyclic selenides **25–27** with an amine group.

The GPx-like activity of open-chain selenide **24** with cyclic selenides **25–27** was compared using the coupled reductase assay^48^ in aqueous medium and also evaluated using the DTT^red^/DTT^ox^ NMR assay in CD_3_OD.^51,52^ The initial reduction rates (*v*_0_) and the times taken for 50% conversion of DTT^red^ to DTT^ox^ in CD_3_OD (*T*_50_) for selenides **24–27** are summarized in Table 8.

**Table 8:** GPx-like Activities of Catalysts **24**–**27** in Water and in CD_3_OD

catalyst	GPx-like activity *v*_0_ (μM·min^–1^)^a^	*T*_50_ (min)^b^
no catalyst	32.20 ± 3.20	>300
**24**	44.20 ± 2.40	10
**25**	59.80 ± 1.10	20
**26**	47.20 ± 4.30	6
**27**	48.2 ± 5.30	65

a Initial reaction rates (*v*_0_) for H_2_O_2_ reduction at 25 °C and pH 7.4 (phosphate buffer).

b Duration for 50% conversion of DTT^red^ to DTT^ox^ in CD_3_OD.

The stereoconfiguration of substituents does not demonstrate any impact on the GPx-like activity of selenides in aqueous medium, unlike in methanol. In aqueous medium, GPx-like activity was observed as **24** < **25** < **26** < **27,** while in methanol, the activity order was observed as **27** < **25** < **24** < **26** (Figure 5).

**Figure 5:**
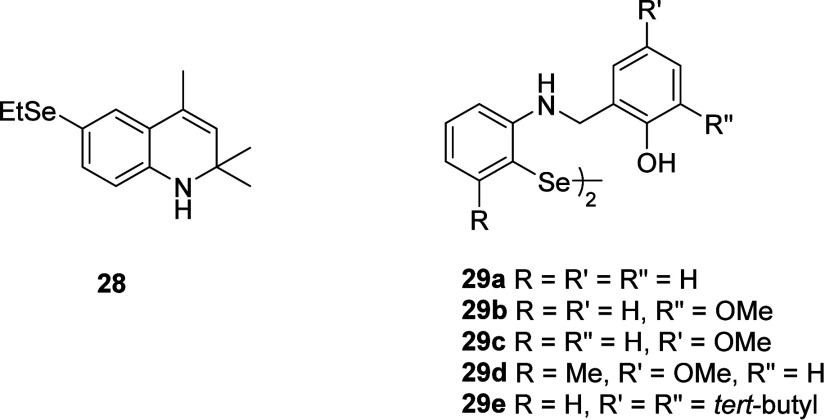
Se analogue of ethoxyquine 28 and diselenides 29a-e..^70^

### 3.5 Aminic Organoselenium Antioxidants by Engman and Kumar et al.

|The RTA activity was assessed in a two-phase model of the lipid peroxidation system in the presence and absence of NAC as a coantioxidant and compared with α-TOH.^53^ The inhibition period (*T*_inh_) and inhibited peroxidation rate (*R*_inh_), measured with and without NAC in the aqueous phase (Table 9).

**Table 9:** NAC (1 mM) Effects the Inhibited Rate (*R*_inh_) and Inhibition Time (*T*_inh_) of Conjugated Diene Formation for Catalysts **28** (20 μM)^a^

			without NAC in aqueous phase		GPx-like activity ν_0_ (μM·min^–1^)^d^
catalyst	*R*_inh_ ^b^ (μM·h^–1^)	*T*_inh_ ^c^ (min)	*R*_inh_ ^b^ (μM·h^–1^)	*T*_inh_ ^c^(min)	
**28**	74	150	70	50	1.26 ± 0.33
α-TOH	25	50	20	40	
Ph_2_Se_2_					0.59 ± 0.21

a The GPx-like activity of catalyst **28** and Ph_2_Se_2_ were assessed by measuring the initial rate of NADPH consumption.

b Rate of peroxidation during the inhibited phase (uninhibited rate ca. 650 μM/h).

c Inhibited phase of peroxidation. Errors correspond to ± SD for triplicate.

d Reduction of H_2_O_2_ (3.75 mM) with PhSH (1 mM) in the presence of various diselenide catalysts (0.1 mM).

The results showed that catalyst **28** exhibited better inhibition of lipid peroxidation with lower regenerability. The GPx-like activity was compared with the Ph_2_Se_2_, used as a reference, and it was found that catalyst **28** exhibited higher activity than Ph_2_Se_2_. Recently, the synthesis of a series of diselenides **29a**–**c** and their GPx-like activity have been described by Kumar and co-workers.^71^ The initial reduction rate (*v*_0_) was measured by the presence of disulfide absorption at 305 nm, as summarized in Table 10.

**Table 10:** GPx-like Activity of Catalysts **29a**–**e** ^a^

catalyst	GPx-like activity *v*_0_ (μM·min^–1^)^a^	catalyst	GPx-like activity *v*_0_ (μM·min^–1^)^a^
Ph_2_Se_2_	24.12 ± 1.86	**29c**	59.42 ± 0.54
**29a**	48.15 ± 2.68	**29d**	51.50 ± 0.04
**29b**	51.84 ± 2.35	**29e**	74.40 ± 3.10

a Catalytic reduction of H_2_O_2_ (3.75 mM) by thiophenol (1 mM) using diselenide catalysts (0.1 mM).

It was observed that di-*tert*-butyl-substituted catalyst **29e** showed maximum thiol oxidation with a peroxide reduction rate of 74.40 ± 3.10 μM min^–1^. The radical scavenging antioxidant activity of diselenides **29a**–**e** was also analyzed by DPPH assay, and it was found that catalyst **29d** showed maximum HAT activity to the DPPH•.

### 3.6 Aminic Organoselenium Antioxidants by Engman and Singh et al.

Previously, we and Engman were continuously involved in the preparation and evaluation of the antioxidant activity of aminic organoselenium compounds. The introduction of a chalcogen atom to the aminic compounds was introduced to design multifunctional antioxidants, behaving as GPx-mimics as well as RTAs.^72^ Earlier, *ortho-*amino-substituted diselenides **30a**–**b** have been reported (Figure 6).^73^ These diselenides were found to exhibit significantly higher GPx-like activity compared to **1** in the coupled reductase assay (Table 11).^48,73^

**Figure 6:**
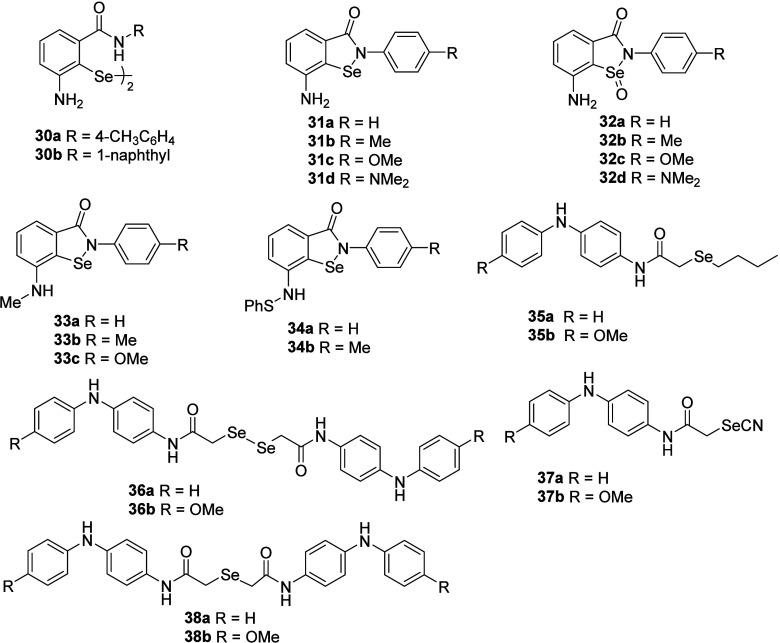
Aminic organoselenium compounds.

**Table 11:** GPx-like Activity of Catalyst **1** and Catalysts **30a**–**b** Were Calculated Using the Initial Consumption Rate of NADPH

catalyst	GPx-like activity *v*_0_ (μM·min^–1^)^a^
**1**	46.50 ± 0.8
**30a**	271.6 ± 5.7
**30b**	133.8 ± 2.7

a Conditions for assay: NADPH (0.2 mM), pH 7.5 with EDTA (1 mM), GSH (1 mM), GR (1.3 unit/mL), H_2_O_2_ (0.80 mM), phosphate buffer (100 mM), and catalysts (20 μM). A correction was applied to the initial rates (ν_0_) for the spontaneous oxidation of GSH (24.3 ± 1.2 mM min^–1^). Errors are reported as standard deviation (±SD) from three replicates.

Recently, various ebselenamines **31a**–**d** and their selenoxide derivatives **32a**–**d** have been reported for the RTA activity and GPx-like activity by our group.^74^ The RTA activity of compounds **30a**–**d** was assessed in a two-phase model of the lipid peroxidation system in the presence of AscOH as a coantioxidant.^53^ Measurements of the inhibition time (*T*_inh_) and inhibited rate of peroxidation (*R*_inh_) were taken in the aqueous phase in the presence and absence of ascorbic acid (AscOH). GPx-like activity was assessed in a coupled reductase assay^48^ and initial rates (ν_0_) calculated for the NADPH consumption are depicted in Table 12.

**Table 12:** AscOH (1 mM) Effects the Inhibited Rate (*R*_inh_) and Inhibition Time (*T*_inh_) of Conjugated Diene Formation for Catalysts **31a**–**d** (40 μM)^a^

	with AscOH		without AscOH		GPx-like activity ν_0_ (μM·min^–1^)^d^	BDE_N–H_ (kcal/mol)	*E* (kcal/mol)
compounds	*R*_inh_ ^b^ (μM·h^–1^)	*T*_inh_ ^c^ (min)	*R*_inh_ ^b^ (μM·h^–1^)	*T*_inh_ ^c^ (min)			
α-TOH	26 ± 2	125 ± 3	28 ± 2	109 ± 2			
**1**	512 ± 5	0	514 ± 4	0	47 ± 1		
**31a**	280 ± 12	0	506 ± 11	0	71 ± 4	81.6	167.6
**31b**	277 ± 12	0	478 ± 7	0	78 ± 3	82.1	165.3
**31c**	15 ± 3	116 ± 6	475 ± 7	0	91 ± 4	82.0	161.8
**31d**	5 ± 1	691 ± 6	11 ± 2	96 ± 5	97 ± 2	82.0	149.6
**32a**					114 ± 5		
**32b**					118 ± 1		
**32c**					116 ± 4		
**32d**					118 ± 2		
control					21 ± 2		

a The GPx-like activity of **1** and catalysts **31a**–**d** and **32a**–**d** were assessed by measuring the initial rate of NADPH consumption.

b Peroxidation rate during the inhibited period (uninhibited rate: ∼544 μM/h).

c Peroxidation’s inhibited period.

d Conditions for assay: NADPH (0.2 mM), pH 7.5 with EDTA (1 mM), GSH (1 mM), GR (1.3 unit/mL), H_2_O_2_ (0.80 mM), phosphate buffer (100 mM), and catalysts (20 μM). Errors are reported as standard deviation (±SD) from three replicates.

From the obtained data, selenoxide catalysts **32a**–**d** exhibited higher GPx-like activity compared to **1** and catalysts **31a**–**d**. The reason for the higher GPx-like activity of **32a**–**d** is that the thiol reduction proceeds much faster than the oxidation, which is the rate-limiting step. A low *R*_inh_ value demonstrated the potent efficacy of the antioxidants in suppressing lipid peroxidation. Catalyst **31d** showed maximum RTA activity, as the strong electron-donating substituent (−NMe_2_) on the *N*-phenyl ring of **31d** may accelerate HAT from the adjacent free -NH_2_ group and improve antioxidant lipophilicity, thereby extending its retention within the lipid layer. The regeneration of AscOH is proposed to occur via a combination of HAT and electron transfer (ET), followed by proton-coupled electron transfer (PCET), where it quenches the peroxyl radicals (LOO•).

The N–H bond dissociation enthalpies (BDE_N–H_) of compounds **31a–d** were measured in the gas phase at the B3LYP/6–311+G­(d) level of theory (Table 12).^30^ Spin density analyses of the radical cations from one-electron oxidation indicate a delocalized electron density in **31a–c** (Figure 7).

**Figure 7:**
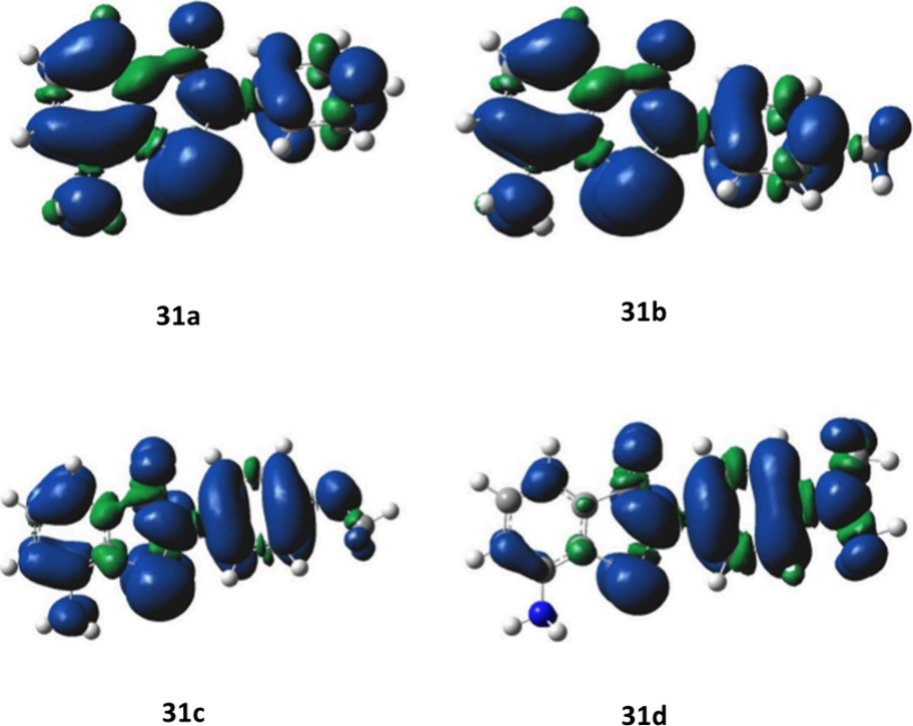
Spin density distributions of the one-electron oxidized radical cations derived from **31a**–**d**.

Further, calculations also revealed that the stability of the generated radical cation was influenced by the substituents. Consistent with this, the spin density was primarily located on the *N*-phenyl ring of compound **31d**, indicating its instability following one-electron oxidation. This confirms the pronounced effect of strong electron-donating groups in **31d** relative to that in **31a–c**. The computed one-electron oxidation energies further demonstrated that **31d** requires less energy (148.8 kcal/mol) for electron removal than **31a–c** (161–166.9 kcal/mol) (Table 12). To rationalize the exceptional antioxidative capacity exhibited by ebselenamine compounds, a nontraditional mechanism has been proposed for regenerable antioxidant **31a** as shown in Scheme 3, involving the transfer of an oxygen atom from LOO• to Se, proceeded by hydrogen abstraction from the −NH_2_ group of selenoxide intermediate **I** to the resulting alkoxyl radical (LO•) in the solvent cage. The AscOH-mediated reduction of aminyl radical selenoxide **II** to aminyl radical selenide **III** is thermodynamically favorable. AscOH, a coreductant in the aqueous phase, regenerated **31a** from **III**. As a consequence, the final product of peroxidation can be an alcohol, not a hydroperoxide. Typical chain-breaking antioxidants halt the autoxidation chain by formally transferring a hydrogen atom to the LOO• radical. Yet, the resulting hydroperoxide needs separate reduction by a supplementary preventive antioxidant. Our novel antioxidants concurrently acted as chain-breaking and peroxide-decomposing antioxidants.

**Scheme 3:**
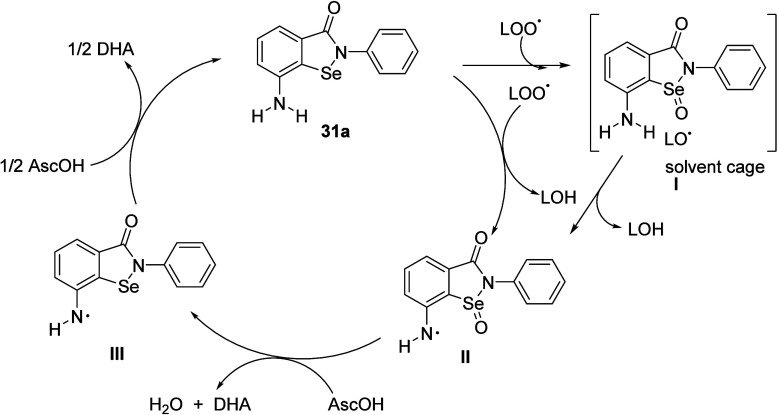
Proposed Mechanism for Ebselenamine Compound **31a**

Later on, the antioxidant properties of *N-*methylated ebselenamines **33a**–**c** were described by coupled reductase assay^48^ and radical quenching activity and regenerability in two phase model system^53^ (Table 13).^75^

**Table 13:** AscOH (1 mM) Effects the Inhibited Rate (*R*_inh_) and Inhibition Time (*T*_inh_) of Conjugated Diene Formation for Catalysts **33a**–**c** (20 μM)^a^

	with AscOH		without AscOH		GPx-like activity ν_0_ (μM·min^–1^)^d^
catalyst	*R*_inh_ ^b^(μM·h^–1^)	*T*_inh_ ^c^ (min)	*R*_inh_ ^b^ (μM·h^–1^)	*T*_inh_ ^c^ (min)	
α-TOH	23 ± 2	125 ± 3	28 ± 2	109 ± 2	
**1**	512 ± 5	0	537 ± 4	0	47 ± 1
**33a**	18 ± 2	224 ± 3	38 ± 4	66 ± 3	83 ± 4
**33b**	12 ± 3	588 ± 6	35 ± 3	92 ± 4	120 ± 3
**33c**	11 ± 3	416 ± 7	30 ± 4	85 ± 3	106 ± 6

a The GPx-like activity of **1** and catalysts **33a**–**c** were assessed by measuring the initial rate of NADPH consumption.

b Peroxidation rate during the inhibited period (uninhibited rate: ∼569 μM/h).

c Peroxidation’s inhibited period.

d Conditions for assay: NADPH (0.2 mM), pH 7.5 with EDTA (1 mM), GSH (1 mM), GR (1.3 unit/mL), H_2_O_2_ (0.80 mM), phosphate buffer (100 mM), and catalysts (20 μM). A correction was applied to the initial rates (ν_0_) for the spontaneous oxidation of GSH (27 ± 1 mM min^–1^). Errors are reported as standard deviation (±SD) from three replicates.

Ebselenamines **33a**–**c** with AscOH outperformed α-TOH and exhibited remarkable radical quenching activity and regenerability. Also, the GPx-like activities of compounds **33a**–**c** were found to be greater than compound **1**. The greater activity might be due to the existence of the (MeNH)-group in the immediate vicinity of the Se atom.

Moreover, the antioxidant properties of *N-*thiophenyl ebselenamines **34a**–**b** were also described using coupled reductase assay and radical quenching activity and regenerability using two phase model system (Table 14).^76^

**Table 14:** AscOH (1 mM) Effects the Inhibited Rate (*R*_inh_) and Inhibition Time (*T*_inh_) of Conjugated Diene Formation for Catalysts **34a**–**b** (20 μM)^a^

	with AscOH		without AscOH		GPx-like activity ν_0_ (μM·min^–1^)^d^
catalyst	*R*_inh_ ^b^ (μM·h^–1^)	*T*_inh_ ^c^ (min)	*R*_inh_ ^b^ (μM·h^–1^)	*T*_inh_ ^c^ (min)	
α-TOH	23 ± 2	96 ± 6	28 ± 2	109 ± 2	
**1**	510 ± 6	0	540 ± 4	0	60.3 ± 2.2
**34a**	14 ± 3	43 ± 6	26 ± 4	30 ± 4	58.4 ± 3.5
**34b**	9 ± 2	88 ± 4	22 ± 3	56 ± 3	112.1 ± 4.6

a The GPx-like activity of **1** and catalysts **34a**–**b** were assessed by measuring the initial rate of NADPH consumption.

b Peroxidation rate during the inhibited period (uninhibited rate: ∼544 μM/h).

c Peroxidation’s inhibited period.

d Conditions for assay: NADPH (0.4 mM), pH 7.5 with EDTA (1 mM), GSH (2 mM), phosphate buffer (100 mM), GR (1.65 unit/mL), H_2_O_2_ (1.60 mM), and catalysts (80 μM). A correction was applied to the initial rates (ν_0_) for the spontaneous oxidation of GSH (6.7 ± 0.30 mM min^–1^). Errors are reported as standard deviation (±SD) from three replicates.

The radical quenching efficiency of antioxidants **34a**–**b** was nearly equivalent to that of α-TOH without AscOH, albeit with a shorter *T*_inh_, whereas, in the presence of AscOH, antioxidants **34a**–**b** showed remarkable radical quenching efficiency. *N-*thiophenyl ebselenamine **34a** demonstrated comparable activity to compound **1,** while catalyst **34b** was found two folds more active than compound **1**. The enhanced activity might be caused by an electron-donating group located on the *N-*phenyl ring.

Very recently, synthesis and GPx-like activity of various diarylamine-based organoselenium compounds **35–38** have been reported using the thiophenol assay.^77^ The initial rates for the disulfide formation are shown in Table 15.

**Table 15:** Catalytic GPx-like Activity of **35–38** Measured by Initial Reduction Rates (ν_0_) Using Thiophenol

catalyst	GPx-like activity ν_0_ (μM·min^–1^)^a^	catalysts	GPx-like activity ν_0_ (μM·min^–1^)^a^
control	2.65 ± 0.84	**36b**	18.12 ± 0.15
*n-*Oct_2_Se_2_	8.19 ± 0.35	**37a**	10.34 ± 0.03
**35a**	18.61 ± 1.42	**37b**	13.24 ± 0.55
**35b**	19.41 ± 0.09	**38a**	18.80 ± 1.36
**36a**	18.08 ± 1.45	**38b**	20.70 ± 0.13

a Conditions for assay: The reactions were carried out at 25 °C in MeOH. Catalyst: 0.01 mM; H_2_O_2_: 3.75 mM, and PhSH: 1 mM. The control experiments were performed under identical conditions in the absence of Se catalysts. A correction was applied to the initial rates (ν_0_) for the spontaneous oxidation of PhSH (2.65 ± 0.84 μM min ^–1^) by H_2_O_2_. Errors correspond to ± SD for triplicate.

The GPx-like activity for catalysts **35–38** was found to be greater than *n-*Oct_2_Se_2_, which was used as a reference. Methoxy-substituted analogues of catalysts **35–38** showed higher GPx-like activity due to their electron-donating nature, as the increased electron density at the Se atom increased the catalytic rate of H_2_O_2_ reduction. Subsequently, it was demonstrated that the GPx-mimics **35–38** were found to be potent antiferroptotic agents. The antiferroptotic activity for compounds **35–38** has been evaluated in cells where GPx4 knockout is induced by 4–OH-tamoxifen using liproxstatin as a standard. Based on some earlier studies^76^ and recent observations,^74,75^ a mechanism has been proposed for the antiferroptotic activities of antioxidant **38a** (Scheme 4).

**Scheme 4:**
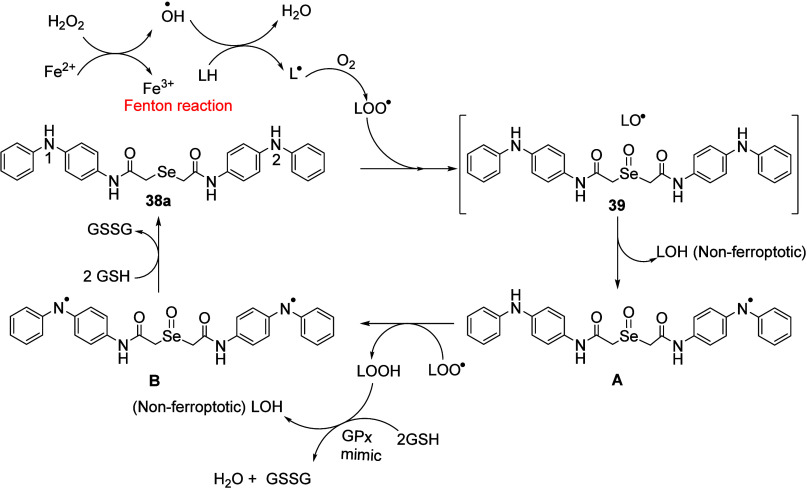
Proposed Mechanism by Which Compound **38a** Inhibits Ferroptosis

Ferroptosis inducers generate free LOO• radicals via the Fenton type reaction to produce the •OH radical. This led to the oxidative damage to lipid molecules (LH) with generation of lipid radical (L•), which then reacted with molecular oxygen (O_2_) to produce ferroptosis. It can be inhibited by small molecules at different molecular steps. The proposed mechanism involves first the transfer of an oxygen atom from LOO• to the Se atom of **38a**, generating the formation of selenoxide **39** and LO• followed by HAT from 1-NH group. In a solvent cage system, LO• abstracted a H atom from the nearby 1-NH group and thus resulted in the formation of selenoxide/aminyl radical intermediate **A** with elimination of LOH. Another LOO• can remove the second H atom from the 2-NH group to produce a diradical intermediate **B**. The resulting LOOH can be reduced into LOH using the GPx mimic **38a** with GSH and thus providing nonferroptotic. Regeneration of **38a** from inetrmediate **B** may be brought by the presence of GSH. Ultimately, LOO• is reduced to LOH and protected cells against ferroptosis. It is also worth mentioning here that the rate-limiting transfer of amine H atom to LOO• facilitated by PCET, a single step bimolecular reaction. In the pursuit of superior synthetic antioxidants, *ortho*-substituted aminic organoselenides **40**, featuring free amine and benzamide groups, demonstrated potent GPx-like and antiferroptotic effects. These compounds protected a TAM-inducible GPx4 knockout cell line from enzyme loss-induced death (Figure 8).^78^

**Figure 8:**
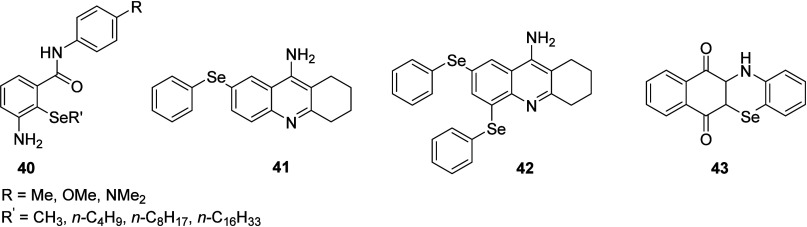
Some organoselenium aminic antioxidants.

In silico molecular docking simulations revealed promising Moldock scores between these antioxidants and human 15-lipoxygenase-2 enzyme. In silico study of human 15-LOX-2 enzyme showed that the binding energy values for antioxidants carrying a selenomethyl group on a small chain demonstrated good affinity. It is therefore both lipid peroxidation and lipoxygenase catalysis may contribute differently to ferroptosis in other cell types depending on lipophilicity. Future studies is required in order to understand how lipid peroxidation is involved particularly in ferroptosis. It is anticipated that the radical-trapping, GPx-like and antiferroptotic activity mechanisms of all organoselenium antioxidants will enable the development of highly efficient inhibitors of ferroptosis that may be useful as therapeutics in diseases caused by lipid peroxidation for future diseases.

Some selenylated analogs **41** and **42** of Tachrine with promising antioxidant effects were found in neutralizing ABTS radicals, scavenging DPPH radicals, and reducing iron ions with the acute oral toxicity.^79^ Selenazidione based naphthoquinonyl organoselenium compound **43** has been studied for photophysical studies (i.e., UV–visible, emission, solvatochromism, and quantum yield) were also screened for antibacterial properties.^80^

## 4 Conclusions and Future Perspectives

The present review highlights a series of simple aminic organoselenium compounds designed to mimic selenoproteins, particularly GPx enzymes. An adjacent aminic moiety significantly boosts the GPx-like activity of these organoselenium catalysts. A precise understanding of the steric and electronic interplay between the Se center and the aminic group during catalysis has provided valuable guidance for designing new organoselenium molecules with improved GPx-mimetic properties.

Some strong evidence is concluded from the above survey of aminic organoselenium compounds that enhanced their antioxidant activity. The presence of strong Se···O/N interactions within selenyl sulfide intermediates impedes the biological efficacy of amine-containing diselenides by facilitating thiol exchange reactions. Therefore, the antioxidant capacity of an organoselenium compound is enhanced by substituents that promote the nucleophilic attack of a thiol on sulfur within its selenenyl sulfide adduct. The higher activity observed for *sec*-amino group compared to *tert*-amino group containing diselenides is attributed to the minimal occurrence of thiol exchange in the former’s selenenyl sulfides. Notably, the catalytic performance of *tert*-amino group compounds was improved by adding substituents that sterically impede the stabilizing Se···N interactions in the selenenyl sulfide state. Furthermore, electron-donating groups at the ortho or para positions of the amine and Se atoms also affected the compounds’ ability to mimic GPx activity. Additionally, the proximity of the aminic group to Se not only enhances GPx-like activity but also facilitates RTA behavior, underscoring the dual functionality of these compounds.

In conclusion, this review highlights the importance of developing novel, simple aminic organoselenium compounds that effectively mimic selenoproteins. The advancements discussed here along with the identified research perspectives are expected to stimulate further innovation in organoselenium chemistry. While significant progress has been made, key challenges and opportunities remain to be addressed. Few promising directions and challenges remain unexplored, which could shape future research in this field as future perspectives: (i) Structural Optimization and Design; (ii) Mechanistic Elucidation; (iii) Biological Applications; (iv) Evaluation Methods; (v) Toxicity and Safety; (vi) Clinical Translation; (vii) Application of Emerging Technologies, e.g., AI-Driven Discovery and CRISPR Screening.
